# The Effect of Prostate Cancer Educational Program on the level of Knowledge and Intention to Screen among Jordanian Men in Amman

**DOI:** 10.31557/APJCP.2020.21.1.211

**Published:** 2020

**Authors:** Ahmad M Saleh, Elturabi Elsayed Ebrahim, Eid Hamed Aldossary, Mariam Awad Mazyad Almutairi

**Affiliations:** *Prince Sattam Bin Abdulaziz University, College of Applied Medical Sciences, Department of Nursing, Alkharj, Riyadh, Saudi Arabia. *

**Keywords:** Prostate cancer, educational program, Jordanian men, level of knowledge, intention to screen

## Abstract

**Purpose:**

The purpose of this study was to examine the effect of prostate cancer educational program on the level of knowledge and intention to screen for prostate cancer among Jordanian men in Amman.

**Methods:**

A quasi-experimental, with nonequivalent control group design was used. 154 participants were randomly assigned to the intervention and control groups. Level of Knowledge and intention to screen were measured at baseline and at 1 month after the application of the prostate cancer educational program. Independent sample t-test was used to analyze the data.

**Results:**

The results showed statistically significant change in the mean knowledge scores (8.7), p < 0.001 and the mean of intention to screen scores (3.71), p < 0.001, after 1 month from the application of the educational program in the experimental group compared to the control group.

**Conclusion:**

Implementing prostate cancer educational programs help enhance knowledge and intention to screen among Jordanian men.

## Introduction

Prostate cancer (PC) has increasingly gained importance as a public health concern that primarily affects older men. It is the second common cause of cancer death in the Western world (Torre et al., 2015). According to the National Center for Health Statistics, there were 679,000 new PC patients worldwide; in the year 2002. The prevalence of PC was estimated to be 19% in the industrialized countries and 5.3% in the developing countries. PC is the most famous type of cancer found in American men, other than skin cancer (National Center for Health Statistics, 2006). Approximately 15,000 PC patients and about 8,000 deaths per annum were reported in the United Kingdom (Kassianos et al., 2016). 

Globally, PC is the sixth leading reason of cancer death in males, and the eleventh leading reason of death from cancer in all groups age (Ferlay et al., 2010; Lozano et al., 2013; Torre et al., 2015). In Jordan, PC is the third most frequently occurring cancer among males, with 218 new cases in 2010 (9.4%) and the third most common cause of death in males about (6.2%) of total deaths in 2010 (Tarawneh et al., 2010).

These statistics indicate that PC Screening (PCS) is essential for protecting men’s health, despite existing problems with screening specificity and sensitivity (Moyer, 2012). If detected early, while the tumor is still confined to the prostate, men remain alive five years post-diagnosis, the survival percentage is 90% compared to 35% for more advanced cancer, early detection remains a key factor in reducing mortality and morbidity (Weinrich et al., 1998). The American Cancer Society (ACS) advises receiving annual digital rectal exams (DREs) and prostate-specific antigen (PSA) tests, starting at age 45 years for at-risk groups comprising individuals with first degree relatives diagnosed with PC at an early age. Others should be screened annually from 50 years onwards (ACS, 2018b). Risk factors for PC include being male, aging, family history (Hx), high-fat product consumption, genetic changes, and obesity. Other potential risk factors such as smoking, being a firefighter, prostatitis, sexually transmitted infections (STI), and vasectomy did not demonstrate as clear relation to PC (ACS, 2018a). Modification of risk factors, intention to screen, and early screening contribute to reducing PC progression (ACS, 2018a; NCI, 2012).

Therefore, patients need accessible and adequate health education regarding these preventive and diagnostic strategies. Studies on patient education designed to raise knowledge and rates of early detection among men show that brief and print-based interventions enhance knowledge of symptoms and risk factors (Taylor et al., 2006; Moyer, 2012), as well as rates of screening (Taylor et al., 2006; Taylor et al., 2016). No studies yet have addressed prostate-related educational program conducted in Jordan. Therefore, it is essential to examine the PC educational program impact on knowledge, and intention to screen among Jordanian men.

*Literature review*

Many studies have demonstrated the effectiveness of different PC educational resources based on the level of knowledge, and intention to screen (Ukoli et al., 2013; Drake et al., 2010; Çapık and Gözüm, 2011; Keane, 2015). Capanna et al., (2015) conducted a pretest/posttest design to assess the impact of a theory-based health education intervention on the awareness of PC and the intention to screen among 454 men in Western Jamaica, aged 40 years and above, utilizing various clinics and hospitals in Western Jamaica, and should not have been previously screened for PC.

The educational intervention was based on constructs from the Transtheoretical Model and the HBM. The educational intervention was administered by a study staff member, using a PowerPoint^®^ presentation which was either displayed on a computer or on printed slides. Upon completing the pre-test, participants observed a PC health education intervention and immediately completed a post-test survey. Findings revealed that there was a significant improvement in PC knowledge, screenings intentions between the pre-test and post-test (p < 0.05) (Capanna et al., 2015). 

In another similar study, Ukoli et al., (2013) employed a single-group, non-randomized education intervention study design to measure the effect of tailored PC education on knowledge and screening among 539 low-income AA men, aged 42 years and above, who did not screen for PC in the last 12 months. Findings revealed that 15 minutes, PC education intervention (providing tailored interaction and a PC brochure) led to improved knowledge, from 13.27±3.51 to 14.95±4.14 (p < 0.001), and screening from (22.1%) to (62.8%). However, Men without a high school diploma recorded the least post-intervention PC knowledge and screening rate, suggestive of the necessity for more than a single education session. The annual free prostate examination can preserve the positive trend observed (Ukoli et al., 2013). 

In the pre-test and post-test longitudinal study, which was conducted by Çapık and Gözüm (2011) to investigate the effect of web-assisted education and reminders on knowledge and early detection behaviors, regarding PCS among 1,744 Turkish men over 40 years of age. The participants were given a web-assisted education and consultation for 6 months, in addition to reminders, for example, booklets, desk calendar, e-mails, and cell phone messages. Alterations in the screening behaviors and the level of knowledge for patients were investigated at 3, and 6 months after the interventions. Through the study, the participants’ prostate examination raised from (9.3%) to (19.1%). The study concluded that web-assisted education and reminders gave positive alterations in the susceptibility and barrier perceptions of participants. Involvement in early detection also increased (Çapık and Gözüm, 2011).

The literature indicated that to enhance the knowledge and intention to screen regarding PC, it is necessary to plan, conduct and provide an effective prostate cancer educational program that may improve the knowledge and intention to screen among men. In Jordan, there are no studies conducted to examine the effect of prostate cancer educational programs on the level of knowledge and intention to screen among Jordanian men in Amman.

## Materials and Methods

The effect of the prostate cancer educational program on the level of knowledge and intention to screen among Jordanian men in Amman was examined using a quasi-experimental, with nonequivalent control group design.

*Sample and Setting*

A convenience sampling technique was used to recruit the participants, who visited Masjids (praying place). The inclusion criteria for participation in the study included (a) men aged 40 years and above (ACS, 2018b; PCC, 2015) who live in Amman; (b) able to read, hear, understand, and speak the Arabic language. Men with a previous diagnosis of PC are excluded from the study, because of possible confounding knowledge of the disease, thus, it is considered as the only exclusion criterion. The sample size was calculated by using G* power 3.0 software (Faul et al., 2007). To have a power of 0.80 with medium effect size, a total sample of 128 participants is required. An expected 25 % attrition rate was added to avoid the risk of bias, which is usually of concern if the rate exceeds 20% (Polit and Beck, 2010). Thus, an additional number of 32 participants was included to give a total of 160 participants in the sample, 80 participants in each group. All study activities and educational program implementation were conducted in Amman, The Capital of Jordan.

*Data Collection and Procedure*

The study method and protocol were reviewed and approved by the ethical committee in the faculty of nursing at the University of Jordan, and the Ministry of Islamic Awqaf Trust Affairs.

Written informed consent was obtained from all participants who agreed to participate in the study. All participants were reviewed by the primary researcher to ensure the eligibility of the participants to participate in the study. After that, the written informed consent was obtained from each participant. Then, the primary researcher collected the data concerning the knowledge, and intention to screen at zero weeks, these data were collected from 209 men for both groups. After that, the primary researcher implemented the prostate cancer educational program for 97 participants. One month after the program application, the primary researcher collected the posttest data from 154 participants.

*Instrumentation*

A structured questionnaire was utilized for collecting the data to achieve the purpose of the study. The questionnaire started with a brief statement concerning the purpose of the study, informed consent, and followed by three parts. Part one is the demographic, which consists of a checklist multiple choice, and gap filling questions type concerning all variables like age, gender, monthly income, and educational level. The second part is The Knowledge of Prostate Cancer and the intention to screen scale.

The knowledge of the PC screening questionnaire. A translated version of the knowledge of the PC screening questionnaire developed by Weinrich et al., (2004), was used to measure participants’ knowledge about PC and PCS. 12 items were used to measure knowledge about PCS limitations, PC symptoms, PC risk factors; side effect from treatment and screening age guidelines. 

An overall knowledge score was computed by totaling the number of correct responses, with a possible range from 0 to 12, and higher scores indicating greater knowledge. Items were tested for internal consistency reliability in the current study and the results revealed that Cronbach’s α coefficient was 0.77, prior to PC educational program, while it was 0.81 for the total scale, post PC educational program.

The intention to screen scale: The translated scale was developed based on the guidelines given by Francis et al., (2004), to measure the generalized intention regarding PCS. The PCS intention scale is composed of three items indicating intention to screen, presented in the Arabic language and measured on a five-point Likert scale (“strongly disagree = 1” to “strongly agree = 5”) with total scores ranging from 3–15, with higher scores indicating a higher degree of intention to screen. Items were tested for internal consistency reliability by Cronbach’s α coefficient which was reported in previous studies to be around 0.95. Items were tested for internal consistency reliability in the current study and the results revealed that

Cronbach’s α coefficient was 0.95 for the total scale, prior to PC educational program, while it was 0.83 for the total scale, post prostate educational program. In addition, content validity was tested in the previous study based on a matrix suggested by Abuadas et al., (2015).

The Permission to use the original and translated questionnaires was obtained from the authors, the translated versions were reviewed by another a group of Jordanian faculty members for proper language use and cultural appropriateness. The questionnaires were pilot tested with 20 participants who met the inclusion criteria of the study. 

The estimated time to complete the questionnaires was 20-30 min. Face validity of the two instruments was assessed by four experts in the area of Prostate Cancer. The results revealed that the two instruments were valid and measuring what was supposed to measure.

*The Prostate Cancer Educational Program*

The prostate cancer educational program took approximately 1-hour educational session consisting of a 30-minute lecture that was conducted by the researcher, a booklet and brochure, that summarized the material provided by an investigator and a 30-minute interactive group discussion. Some individualized sensitive questions were answered individually. The brochure “Prostate Cancer: What you should know about prostate cancer” was adopted from Medical Cancer Center. This educational module was written in the Arabic language and was derived from relevant literature in Evidence-Based Practice (ACS, 2018a).

The brochure was developed and reviewed by a multidisciplinary team of an oncologist, oncology nurse educator, clinical nurse specialist, laboratory technician, and radiologist. Booklet educational material was developed by the researcher to complement the information missed in the brochure. Both booklet and brochure materials were evaluated by a panel of experts, including two urologists, consultant clinical oncology, and one nurse, with over 7 years’ experience in oncology critical care, to ensure the adequacy of the information that was provided to the participants. 

The educational booklet and the brochure covered the information related to the overview of the prostate gland, an overview of neoplasm, risk factors for developing PC, PCS, signs, and symptoms of PC, diagnosis, grading scale for diagnosing PC, treatments and its side effects, follow up caring and preventive measures.

*Data Analysis*

The Statistical Package for the Social Science (SPSS) software, version 21 was used to analyze the study data (International Business Machines Corporation, 2012). Descriptive statistics were used to describe the sample characteristics. Independent sample t-test was used to assess whether or not there were statistically significant differences in the level of knowledge, and adherence intention to healthy lifestyle scores between experimental and control group after the implementation of the prostate cancer educational program.

## Results

*Sample Characteristics*

One hundred and three participants were involved in the study analysis, indicating a response rate of 74%. The mean age of participants was 53.1 years (SD = 9.52) and ranged between 40-years old and 78-years old years. Most of the participants (94.8%) were married, in addition, (40.3%) had a baccalaureate level of education. Table 1. Concerning those participants who did not complete the entire study (N = 27) were like those participants who completed the entire study.

*Level of Knowledge and Intention to Screen*

The results of the current study showed that the change in the mean knowledge scores 8.7, p <0.0001 was statistically significant at 1 month after the application of the program in the experimental group compared to the control group. In addition, the mean of intention to screen 3.71, p < 0.0001 was found to be statistically significant in the experimental group compared to the control group after one month of the prostate educational program implementation.

Findings of the effect of the prostate cancer educational program on the levels of knowledge, and intention to screen are presented in Table 2. The change in the mean scores between groups, the p-value, and t value are reported in this Table.

**Table 1:** Sample Characteristics; Mean, Standard Deviation, and Percentage of participants (N= 103)

Variables (Mean, SD)	N (%)
Age (53.1, 9.52)	
Marital status	
Single	4 (2.6)
Married	146 (94.8)
Divorced/widowed	4 (2.6)
Educational level	
Primary	18 (11.7)
Secondary	58 (37.7)
Diploma	5 (3.2)
Baccalaureate	62 (40.3)
Graduate	11 (7.1)

SD, Standard Deviation; JD, Jordanian Dinar

**Table 2:** Independent Sample t-test on the Level of Knowledge and Intention to Screen Acquisition between Both Groups

Variable	Experimental group (N = 76)	Comparison group (N = 78)		
	M (SD)	M (SD)	t	P value
Knowledge	8.7 (2.422)	4.56 (2.71)	9.972	0
Intention to screen	3.71 (0.77)	3.27 (0.88)	3.271	0.001

SD, Standard Deviation; M, Mean.

## Discussion

The results in the current study indicated that the knowledge among Jordanian men in Amman significantly improved at one month after the application of the prostate cancer educational program. This finding is congruent with the results of earlier research studies that examined the effectiveness of the educational intervention at improving knowledge. For instance, a controlled trial conducted by Wilt et al., (2000), indicated that educational pamphlet enhanced knowledge for men who experienced the educational pamphlet than men in the control group. Other studies have compared various types of educational interventions with their efficacy at enhancing knowledge. Gattellari and Ward (2005), used a pamphlet, a booklet, or a video to educate 421 men, and then, initiated contact 1 week later for follow up. While all interventions significantly increased knowledge, the percentage correct on a post-test, was significantly greater among men who received the booklet, than it was for men who viewed only the video or read the leaflet (p < 0.001). However, Partin et al., (2004), observed that a pamphlet and a video were equally effective. 

Lastly et al., (2000), compared an illustrated pamphlet to a traditional pamphlet and noticed that both increased the level of knowledge, but neither increased the real usage of PSA testing. Similarly, other investigators reported a high knowledge score about PC and PCS, among respondents, after receiving the educational intervention (Drake et al., 2010; Keane, 2015; Ivlev et al., 2018). The significant effect of the prostate cancer educational program on knowledge may be attributed to many factors. The systematic education which included a combination of verbal information, brochure, and booklet help improve participants knowledge. The educational program was standardized and appropriate to the individual in terms of gender, age, Jordanian culture, and socioeconomic factors. The previous factors have an important impact on the ability of individuals to learn (Drake et al., 2010). In addition, the using of open discussion during the application of the prostate cancer educational program and giving written information may have contributed to the success of the intervention. Such approaches have been recognized as being important when performing patient education sessions (Gökce et al., 2017).

Regarding the effect of the prostate cancer educational program on intention to screen, the results of the current study showed that the intention to screen variable improved significantly 1 month after the application of the educational program in the experimental group compared to the comparison group. This finding is congruent with the finding of the previous research studies (Odedina et al., 2014; Keane, 2015; Ivlev et al., 2018), The finding of this study suggests that PCS intention may be influenced by PC educational program. This explanation was supported by evidence in previous literature, which showed a statistically significant increase in intention to screen, after an educational intervention (Odedina et al., 2014; Capanna et al., 2015).

*Limitations*

Convenience sampling as well as limiting the study to the capital of Jordan posed a problem for the generalizability of the findings to all Jordanian men. Short follow-up period to measure the concept of knowledge and intention to the screen were another limitation. Intention to the screen is needed to be measured over a long period of time. The use of self-reported behavior tool were other limitations of the present study.

*Conclusion and Recommendations*

The application of this study in practice may help improve the knowledge and the intention to screen among Jordanian men in Amman. Conducting research exploring the effect of prostate cancer educational program on the level of knowledge and intention to screen for prostate cancer may provide a basis for conducting other studies which can address the gap and the limitations of the present study. The findings from this study deserve to be replicated using a larger and more heterogeneous randomly selected sample.

The effect of the prostate cancer educational program on knowledge and intention to the screen were still of reasonable magnitude 1 month after the program application. Further research is necessary to measure long-term time after the application of the prostate cancer educational program. Findings of this study confirm the importance of the cardiac educational program in improving the knowledge and intention to screen.

## References

[B1] Abuadas MH (2015). Jordanian men’s health beliefs, intentions, and behaviors regarding prostate cancer screening.

[B2] American Cancer Society (ACS) (2018a). Prostate Cancer Overview.

[B3] American Cancer Society (ACS) (2018b). Prostate Cancer Prevention and Early Detection.

[B4] Capanna C, Chujutalli R, Murray S (2015). Prostate cancer educational intervention among men in Western Jamaica. Prev Med Rep.

[B5] Çapık C, Gözüm S (2011). Development and validation of health beliefs model scale for prostate cancer screenings (HBM-PCS): Evidence from exploratory and confirmatory factor analyses. Eur J Oncol Nurs.

[B6] Drake BF, Shelten R, Gilligen T, Allen JD (2010). A church-based intervention to promote informed decision making for prostate cancer screening among African American men. J Natl Med Assoc.

[B7] Faul F, Erdfelder E, Lang A, Buchner A (2007). G*Power 3: a flexible statistical power analysis program for the social, behavioral and biomedical sciences. Behav Res Methods.

[B8] Ferlay J, Shin HR, Bray F (2010). Estimates of worldwide burden of cancer in 2008: GLOBOCAN 2008. Int J Cancer.

[B9] Gattellari M, Ward JE (2005). A community-based randomized controlled trial of three different educational resources for men about prostate cancer screening. Patient Educ Couns.

[B10] Gökce MI, Wang X, Frost J (2017). Informed decision making before prostate-specific antigen screening: Initial results using the American Cancer Society (ACS) Decision Aid (DA) among medically underserved men. Cancer.

[B12] Ivlev I, Jerabkova S, Mishra M, Cook LA, Eden KB (2018). Prostate cancer screening patient decision aids: A systematic review and meta-analysis. Am J Prev Med.

[B13] Kassianos AP, Raats MM, Gage H (2016). An exploratory study on the information needs of prostate cancer patients and their partners. Health Psychol Res.

[B15] Lozano R, Naghavi M, Foreman K (2013). Global and regional mortality from 235 causes of death for 20 age groups in 1990 and 2010: a systematic analysis for the Global Burden of Disease Study 2010. Lancet.

[B16] Moyer V (2012). Screening for prostate cancer: US preventive services task force recommendation statement. Ann Intern Med.

[B19] Odedina F, Awoyemi OO, Pressey S (2014). Development and assessment of an evidence-based prostate cancer intervention programme for black men: The WORD on prostate cancer video. Asia Pac J Clin Oncol.

[B20] Partin MR, Nelson D, Radosevich D (2004). A randomized trial examining the effect of two prostate cancer screening educational interventions on patient knowledge, preferences, and behaviors. J Gen Intern Med.

[B23] Schapira MM, VanRuiswyk J (2000). The effect of an illustrated pamphlet decision-aid on the use of prostate cancer screening tests. J Fam Pract.

[B25] Taylor KL, Davis JL, Turner RO (2006). Educating African American men about the prostate cancer screening dilemma: a randomized intervention. Cancer Epidemiol Biomarkers Prev.

[B27] Torre LA, Bray F, Siegel RL (2015). Global cancer statistics, 2012. CA Cancer J Clin.

[B28] Ukoli FA, Patel K, Hargreaves M (2013). A tailored prostate cancer education intervention for low-income African Americans: Impact on knowledge and screening. J Health Care Poor Underserved.

[B29] Weinrich SP, Seger R, Miller BL (2004). Knowledge of the limitations associated with prostate cancer screening among low-income men. Cancer Nurs.

[B30] Weinrich SP, Weinrich MC, Boyd MD, Atkinson C (1998). The impact of prostate cancer knowledge on cancer screening. Oncol Nurs Forum.

[B31] Wilt TJ, Paul J, Murdoch M (2000). Educating men about prostate cancer screening A randomized trial of a mailed pamphlet. Int J Clin Pract Suppl.

